# Association of all Cause and Cause-Specific Mortality With Hearing Loss Among US Adults: A Secondary Analysis Study

**DOI:** 10.3389/ijph.2022.1604785

**Published:** 2022-05-17

**Authors:** Yiran Cui, Yan Yan

**Affiliations:** Department of Epidemiology and Medical Statistics, Xiangya School of Public Health, Central South University, Changsha, China

**Keywords:** mortality, hearing loss, all-cause, cause-specific, US adults, prospective cohort study

## Abstract

**Objectives:** Previous research revealed the relationship between hearing loss (HL) and all cause mortality. The aim of this study was to determine the association between HL and all causes and cause-specific mortality based on US adults.

**Methods:** Data were obtained by linking National Health Interview Survey (NHIS) (2004–2013) with linkage to a mortality database to 31 December 2015. HL were categorized into four groups: good hearing, a little hearing difficulty, a lot of hearing difficulty, profoundly deaf. The relationship between HL and mortality risk was analyzed using Cox proportional hazards regression model.

**Results:** Compared with the reference group (Good), those who had light or moderate hearing problems were at an increased risk of mortality for all causes (A little trouble—HR: 1.17; 95% confidence interval [CI]: 1.13 to 1.20; A lot of trouble—HR: 1.45; 95% CI: 1.40–1.51); deaf—HR: 1.54; 95% CI: 1.38–1.73) respectively.

**Conclusion:** In addition, those in the deaf category have the highest risk of death from all causes and cause-specific cancer. More older adults are associated with an increased risk of all-cause mortality in American adults.

## Introduction

Hearing loss (HL) is not just one of the causes of disability, but also affects 360 million people in the world [1]. The most recent WHO estimate suggests that approximately 466 million people (or 6.1% of the world’s population) were living with disabling hearing loss in 2018 [2]. HL is also a common social problem and one of chronic diseases in the US [3]. As the third most chronic disability in the US, HL is estimated to affect 29 million Americans. The adult population of HL accounts for approximately 16.8% of the US population. Its prevalence also has risen significantly and increases with age [4–6]. As the population age and noise exposure increases, the incidence of HL may be also growing [3, 7, 8]. The Global Burden of Disease study estimated that the population with hearing loss increased from 1.2 billion (17.2%) in 2008 to 1.4 billion (18.7%) in 2017 [9]. The prevalence of hearing loss is rising year by year [10]. The prevalence of HL among US adults over the age of 65 ranges from 30% to 83% [11, 12]. With the aging population in the US increasing, the number of people suffering from HL is also increasing, which may gradually become a public health problem [13].

Researchers have concluded that there is a relationship between HL and increased mortality risks [14–17]. A recent study showed that older people with severe HL have a 20% increased risk of death compared to people with normal hearing [18]. In addition, a number of studies have shown that HL has a significant negative impact on the health status, social isolation and physical functions of the older adults [19–27]. One of the studies presented that HL can also cause communication difficulties, which not only affect work efficiency, but also negatively affect cognition and emotions [19–24]. HL closely interacts with health. HL can cause many problems, and communication barriers that accompany HL may lead to disability and psychological inferiority, which results in lower self-esteem and even worsen mental health [25, 26]. At the same time, a recent study also found a longitudinal relationship between HL and depression [27].

There are also several studies have found that HL is associated with all-cause mortality and other cause-specific mortality, such as deaths due to influenza and pneumonia disease or chronic lower respiratory disease (CLRD) [28, 29]. Result from evidence suggests that viral and pneumonia infection could be one factor of HL [28]. While most studies are limited to the association of hearing loss with all-cause mortality, our study analyzed all-cause mortality and further discussed hearing loss and cause-specific mortality. In addition, National Health Interview Survey (NHIS) has received approval from the National Center for Health Statistics (NCHS) Research Ethics Review Board, which is based on a secondary analysis of publicly available, de-identified data. The aim of this article is to analyze hearing loss on cause-specific mortality (CVD; heart disease; and stroke; CLRD; cancer; diabetes; Alzheimer’s disease; influenza and pneumonia; and accidents).

## Methods

### Data Source

The data for this study comes from the National Health Interview Survey (NHIS) (2004–2013) in the United States, which is a representative, stratified, multistage probability survey. The US Centers for Disease Control and Prevention and the NCHS conducted annual household interview surveys through the Census Bureau and collected the basic health status of family members. Participants are members of the US population living in different communities. The specific design and method are all in NCHS (www.cdc.gov/nchs/nhis/about_nhis.htm; accessed 7 January 2018). The data is public and in compliance with the ethical board review of the corresponding author’s institution.

### Study Participants

This study included representative US adult data collected in every household from 2004 to 2013. The complete reported hearing loss data was available for a total of 493,036 participants over 18 years of age who met the criteria for this study. Among them, 37,048 were excluded because of missing data on diabetes (*n* = 200), missing data on smoking and drinking (*n* = 8,524), missing data on physical activity (*n* = 11,119) and missing data on potential covariates (personal variables, lifestyle factors or chronic health conditions; *n* = 17,205), resulting in the final analytical sample of 455,988 adults.

### Study Outcomes

The mortality rate is recorded by the National Death Index (NDI). It has showed that the accuracy of all-cause mortality and cause-specific mortality is consistent with NDI records [30, 31]. Cause of death is coded using the International Classification of Diseases (ICD-10) In addition to all-cause mortality, there are mainly outcomes of cause-specific mortality (CVD; heart disease; and stroke; CLRD; cancer; Diabetes; Alzheimer’s disease; Influenza and pneumonia; and accidents). See Supplementary Table SA for the ICD-10 codes.

### Study Exposure

Hearing loss status was ascertained at the initial interview via two binary coded (i.e., yes/no) items: 1) Can you hear what other people are saying when you talk to them. 2) Do family members and friends think you have difficulty hearing. Those participants responding “Yes” to the first item and “No” the second item were classified as good. Those participants responding “No” to both questions were classified as a little hearing difficulty. Those participants responding “No” to the first item and “Yes” the second item were classified as a lot of hearing difficulty. In the present study, the participants were divided according to their general hearing status, ICD-10 codes were used by the NCHS to classify hearing status as follows: as follows: 1) good hearing, 2)a little hearing difficulty, 3)a lot of hearing difficulty, 4)profoundly deaf.

### Covariates

Information on covariates about sociodemographic factors and lifestyle factors at baseline was obtained via examination and questionnaire-based interviews: (1) Sociodemographic factors: age (1∼18–45, 2∼45–65, 3∼65+), sex (1∼male, 2∼female), educational level (1∼Less than high school degree, 2∼High school degree, 3∼More than high school degree) (2) Lifestyle factors as follows: Alcohol intake 1∼Lifetime abstainer, 2∼Former drinker 3∼Current drinker), Smoking status (1∼Never cigarette, 2∼Former cigarette, 3∼Current cigarette), BMI (1∼<25, 2∼25–30, 3∼≥30). In addition, we included physical activity, hypertension, diabetes, stroke, coronary heart disease (CHD), or cancer. Age, sex, race, education level, income, alcohol intake, smoking status, and BMI all were treated as categorical variables.

### Statistical Analyses

Descriptive statistics were used to describe the distribution of parameters and baseline characteristics of US participants’ HL. We used the *χ* ^2^ test to assess the baseline characteristics of participants in the four levels (good, a little trouble, a lot of trouble, deaf) of hearing loss. Chi-square independence test is used to check the classification difference of the weighted percentage of hearing loss. Among this, HL mortality is divided into three age groups according to the NHIS population: 18–45 years old, 45–65 years old, and >65 years and older. We used a multivariate Cox proportional hazards regression model with proportional assumptions to examine the hazard ratios (HR) with 95% CIs for all cause and cause-specific mortality for a little trouble hearing, a lot of trouble hearing, and deaf. To assess the different potential confounding effects on the relationship between levels of HL and mortality, we developed four models by incorporating three sets of covariates into the model in turn. We also conducted a subgroup analysis for all special cause mortality rates at the same time. In order to test the stability of the results, we conducted sensitivity analyses. Including participants with chronic diseases had little effect on risk estimates of all-cause mortality were excluded and all deaths within 2 years of follow-up were excluded.

All data analyses were weighted and conducted using STATA version 12.0 (Stata Corp, College Station, TX, United States). A two tailed *p* < 0.05 was considered to be statistically significant.

## Results

### Population Characteristics

Among 455,988 US adults, there are 380,233 in good status, 62,491 are regarded as a little trouble hearing, 12,044 are a lot of trouble hearing, and 1,220 are in the deaf category. Of these participants, those with hearing loss were more likely to be older, men, Non-Hispanic White, more than a high school degree, coming from a middle income status, physically inactive, current drinkers. Table 1 shows the baseline characteristics of hearing loss among participants aged 18 years and older. Each baseline feature has statistically significant differences in the four categories of HL (*p* < 0.001).

**TABLE 1:** Baseline Characteristics of Hearing loss Among Participants Aged 18 Years and Older, (United States, 2004–2013).

Characteristics	Overall	Good	Little trouble	A lot of trouble	Deaf
	(N = 455,988)	(*n* = 380,233)	(*n* = 62,491)	(*n* = 12,044)	(*n* = 1,220)
Age group (years)					
18–45	222,402 (51.1)	206,571 (56.6)	14,198 (24.9)	1,391 (12.7)	242 (22.6)
45–65	143,570 (32.4)	117,087 (31.3)	22,891 (39.8)	3,294 (30.5)	298 (24.9)
65-	90,016 (16.4)	56,575 (12.1)	25,402 (35.3)	7,359 (56.9)	680 (52.5)
Sex					
Male	202,486 (48.7)	162,696 (46.9)	32,618 (57.3)	6,580 (60.7)	592 (54.9)
Female	253,502 (51.3)	217,537 (53.1)	29,873 (42.7)	5,464 (39.3)	628 (45.1)
Race					
Hispanic	77,826 (12.6)	71,054 (13.8)	5,733 (6.5)	894 (4.9)	145 (8.5)
Non-Hispanic White	288,763 (70.9)	229,087 (68.3)	48,762 (83.8)	9,977 (87.7)	937 (82.1)
Non-Hispanic Black	66,797 (11.6)	59,915 (12.6)	5,980 (6.7)	811 (4.7)	91 (5.6)
Non-Hispanic Other	22,602 (4.9)	20,177 (5.3)	2016 (3.1)	362 (2.7)	47 (3.9)
Education level					
Less than high school degree	86,014 (16.2)	68,481 (15.4)	13,443 (18.5)	3,678 (27.8)	412 (29.9)
High school degree	126,684 (28.4)	103,341 (27.8)	19,141 (31.4)	3,810 (32.8)	392 (33.6)
More than high school degree	243,290 (55.4)	208,411 (56.8)	29,907 (50.1)	4,556 (39.4)	416 (36.5)
Income					
Low	74,336 (12.2)	62,776 (12.4)	9,279 (11.2)	2,048 (13.1)	233 (14.0)
Middle	232,707 (49.6)	190,776 (48.7)	33,871 (52.5)	7,301 (59.8)	759 (62.5)
High	148,945 (38.2)	126,681 (38.9)	19,341 (36.4)	2,695 (27.1)	228 (23.4)
BMI (kg/m^2^)					
<25	181,028 (39.8)	154,804 (41.1)	21,151 (32.4)	4,535 (35.2)	538 (43.8)
25–30	159,252 (35.1)	131,401 (34.6)	23,105 (37.6)	4,363 (37.3)	383 (32.7)
>30	115,708 (25.2)	94,028 (24.3)	18,235 (30.0)	3,146 (27.6)	299 (23.5)
Physical activity					
Yes	193,133 (44.5)	166,508 (45.9)	23,086 (39.3)	3,215 (28.3)	324 (27.3)
No	262,855 (55.5)	213,725 (54.1)	39,405 (60.7)	8,829 (71.7)	896 (72.7)
Smoking status					
Never cigarette	257,463 (56.5)	223,799 (59.0)	27,893 (44.0)	5,128 (40.3)	730 (52.6)
Former cigarette	100,580 (22.2)	74,762 (19.7)	20,873 (34.1)	4,880 (39.7)	413 (31.3)
Current cigarette	97,945 (21.3)	81,672 (21.3)	13,725 (21.9)	2,529 (20.0)	230 (16.1)
Alcohol intake					
Lifetime abstainer	104,467 (21.7)	88,918 (22.4)	12,014 (17.3)	3,090 (23.2)	445 (35.2)
Former drinker	70,739 (14.7)	52,902 (13.1)	14,074 (21.4)	3,465 (28.9)	298 (23.7)
Current drinker	280,782 (63.6)	238,413 (64.5)	36,403 (61.3)	5,489 (47.8)	447 (41.1)
Physician-diagnosed disease					
Hypertension					
Yes	325,811 (26.6)	95,007 (23.1)	28,263 (43.0)	6,351 (51.6)	556 (44.0)
No	130,177 (73.5)	285,226 (76.9)	34,228 (57.0)	5,693 (48.4)	664 (56.0)
Diabetes					
Yes	37,187 (7.5)	26,132 (6.2)	8,772 (13.3)	2082 (18.0)	201 (17.5)
No	418,801 (92.5)	354,101 (93.8)	53,719 (86.7)	9,962 (82.0)	1,019 (82.5)
CHD					
Yes	20,615 (4.2)	12,111 (2.9)	6,347 (9.8)	1979 (17.0)	178 (14.2)
No	435,373 (95.8)	368,122 (97.1)	56,144 (90.3)	10,065 (83.0)	1,042 (85.8)
Stroke					
Yes	12,931 (2.5)	8,374 (1.8)	4,122 (5.4)	1,431 (10.9)	140 (10.0)
No	443,057 (97.5)	372,543 (98.3)	58,700 (94.6)	10,716 (89.1)	1,098 (90.0)
Cancer					
Yes	35,427 (7.5)	23,570 (5.9)	9,202 (14.3)	2,448 (20.4)	207 (17.7)
No	420,561 (92.6)	356,663 (94.1)	53,289 (85.7)	9,596 (79.6)	1,013 (82.4)

CHD, coronary heart disease.

### Hearing Loss and All Cause, Cause Specific Mortality

**FIGURE 1:**
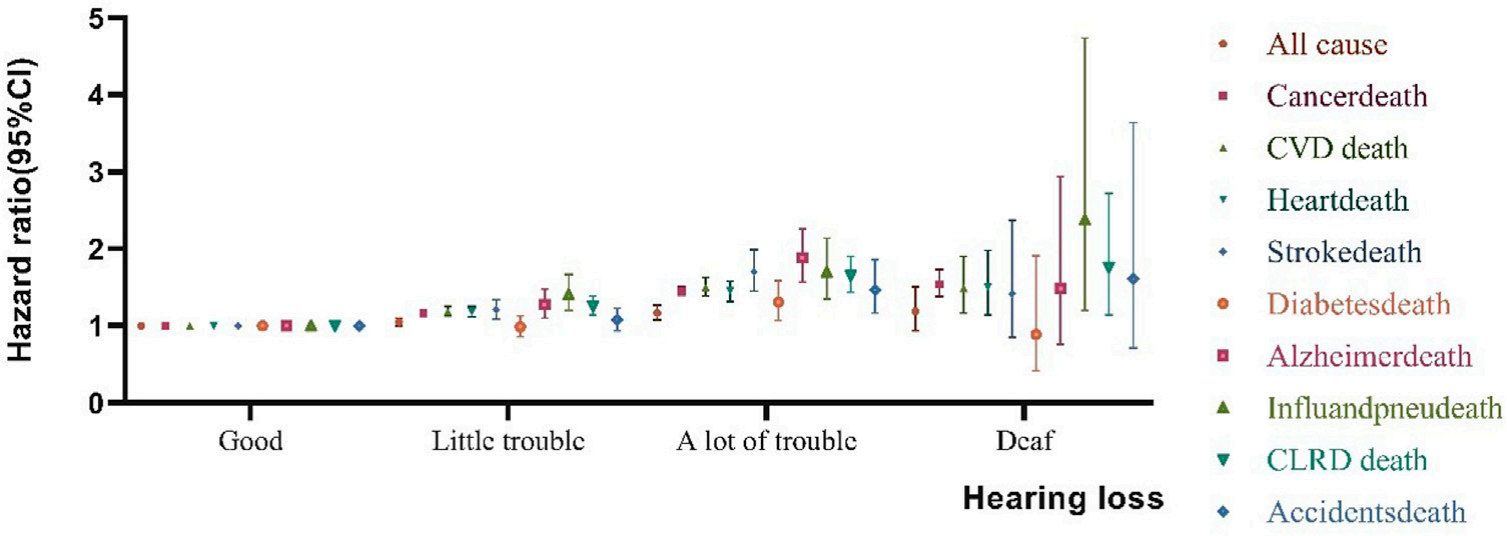
Association between Hearing loss and all cause and specific-cause mortality. Effect estimates are from the fully adjusted Model 4 that includes the covariates of age, sex, education, physical activity, smokers, alcohol intakes, body mass index, hypertension, diabetes, cancer, stroke, and coronary heart disease. Whiskers represent 95% confidence intervals, (United States, 2004–2013).

Table 2 displays the HR of all cause and specific-cause mortality caused by HL. After several years of 479, 856 follow-up, 59,791 participants died from all causes, 13,488 from CVD deaths (10,418 deaths related to heart disease and 3,070 deaths related to stroke disease), 14,317 from cancer deaths, 3,175 from chronic lower respiratory diseases, 2,490 from accidents, 1,478 from Alzheimer’s disease, 1,804 from diabetes, 1,133 from influenza and pneumonia. In unadjusted Model 1, HL had a greater risk of all cause, CVD disease, Alzheimer’s disease, and influenza and pneumonia disease. In comparison to those good hearing, the HR of all-cause mortality were higher in little trouble hearing 2.70 (95% CI, 2.63–2.77) a lot of trouble hearing 5.46 (95% CI, 5.24–5.69) and deaf 5.29 (95% CI, 4.72–5.94). After adjusting the covariates for all-cause mortality in Model 4, the risk of death for people with a little trouble hearing decreased from 2.70 to 1.17. A large number of HL adults have a significant reduction from 5.46 to 1.45, for deaf adults, from 5.29 to 1.54. In addition, after adjusting from model 1 to model 2 (age), the risk of mortality from all nine specific-causes of disease has been greatly reduced. Similar associations were observed between HL and all cause and specific-cause mortality (Figure 1).

**TABLE 2:** Hazard ratios and 95% Confidence Interval of hearing loss for All-Cause and Cause-Specific Mortality, (United States, 2004–2013).

Outcome	Number of deaths	Model 1		Model 2		Model3		Model4	
		HR (95%CI)	*p*	HR (95%CI)	*p*	HR (95%CI)	*p*	HR (95%CI)	*p*
All cause	59,791								
Good	38,956	1 (reference)	0.00	1 (reference)	0.00	1 (reference)	0.00	1 (reference)	0.00
Little trouble	15,197	2.70 (2.63–2.77)	0.00	1.32 (1.29–1.35)	0.00	1.21 (1.18–1.24)	0.00	1.17 (1.13–1.20)	0.00
A lot of trouble	5,156	5.46 (5.24–5,69)	0.00	1.93 (1.86–2.00)	0.00	1.58 (1.53–1.65)	0.00	1.45 (1.40–1.51)	0.00
Deaf	482	5.29 (4.72–5.94)	0.00	2.13 (4.75–4.90)	0.00	1.66 (1.48–1.85)	0.00	1.54 (1.38–1.73)	0.00
Cancer death	14,371	Model 1		Model 2		Model 3		Model 4	
Good	9,932	1 (reference)	0.00	1 (reference)	0.00	1 (reference)	0.00	1 (reference)	0.00
Little trouble	3,377	2.37 (2.27–2.48)	0.00	1.22 (1.17–1.28)	0.00	1.11 (1.06–1.17)	0.00	1.05 (1.00–1.10)	0.00
A lot of trouble	980	4.08 (3.78–4.41)	0.00	1.52 (1.41–1.64)	0.00	1.29 (1.19–1.39)	0.00	1.17 (1.08–1.27)	0.00
Deaf	82	3.63 (2.85–4.63)	0.00	1.55 (1.23–1.95)	0.00	1.29 (1.02–1.64)	0.00	1.19 (0.94–1.51)	0.00
CVD death	13,488	Model 1		Model 2		Model 3		Model 4	
Good	8,374	1 (reference)	0.00	1 (reference)	0.00	1 (reference)	0.00	1 (reference)	0.00
Little trouble	3,653	3.09 (2.95–3.25)	0.00	1.38 (1.31–1.44)	0.00	1.24 (1.18–1.31)	0.00	1.19 (1.13–1.25)	0.00
A lot of trouble	1,351	6.81 (6.31–7.34)	0.00	2.12 (1.98–2.27)	0.00	1.68 (1.56–1.82)	0.00	1.50 (1.39–1.63)	0.00
Deaf	110	6.17 (4.91–7.76)	0.00	2.28 (1.84–2.82)	0.00	1.62 (1.27–2.07)	0.00	1.49 (1.17–1.90)	0.00
Heart death	10,418	Model 1		Model 2		Model 3		Model 4	
Good	6,477	1 (reference)	0.00	1 (reference)	0.00	1 (reference)	0.00	1 (reference)	0.00
Little trouble	2,835	3.12 (2.96–3.30)	0.00	1.40 (1.32–1.48)	0.00	1.24 (1.17–1.31)	0.00	1.18 (1.12–1.25)	0.00
A lot of trouble	1,019	6.66 (6.13–7.24)	0.00	2.12 (1.95–2.30)	0.00	1.62 (1.48–1.77)	0.00	1.45 (1.32–1.58)	0.00
Deaf	87	6.28 (4.84–8.13)	0.00	2.32 (1.82–2.96)	0.00	1.64 (1.24–2.16)	0.00	1.50 (1.14–1.98)	0.00
Stroke death	3,070	Model 1		Model 2		Model 3		Model 4	
Good	1897	1 (reference)	0.00	1 (reference)	0.00	1 (reference)	0.00	1 (reference)	0.00
Little trouble	818	3.00 (2.71–3.32)	0.00	1.31 (1.19–1.45)	0.00	1.26 (1.14–1.40)	0.00	1.21 (1.09–1.34)	0.00
A lot of trouble	332	7.29 (6.25–8.51)	0.00	2.20 (1.88–2.57)	0.00	1.92 (1.64–2.24)	0.00	1.70 (1.45–1.99)	0.00
Deaf	23	5.83 (3.52–9.66)	0.00	1.94 (1.17–3.22)	0.01	1.57 (0.94–2.62)	0.08	1.42 (0.85–2.37)	0.18
Diabetes death	1,804	Model 1		Model 2		Model 3		Model 4	
Good	1,029	1 (reference)	0.00	1 (reference)		1 (reference)		1 (reference)	0.00
Little trouble	430	2.43 (2.13–2.78)	0.00	1.21 (1.06–1.37)	0.01	1.01 (0.88–1.16)	0.87	0.99 (0.86–1.13)	0.85
A lot of trouble	156	5.50 (4.53–6.68)	0.00	2.04 (1.69–2.46)	0.00	1.42 (1.16–1.72)	0.00	1.31 (1.07–1.59)	0.01
Deaf	9	3.40 (1.62–7.15)	0.00	1.35 (0.68–2.69)	0.42	0.93 (0.43–1.99)	0.85	0.89 (0.42–1.91)	0.77
Alzheimer death	1,478	Model 1		Model 2		Model 3		Model 4	
Good	872	1 (reference)	0.00	1 (reference)	0.00	1 (reference)	0.00	1 (reference)	0.00
Little trouble	417	3.43 (2.98–3.95)	0.00	1.26 (1.10–1.45)	0.00	1.29 (1.11–1.49)	0.00	1.28 (1.10–1.48)	0.00
A lot of trouble	177	8.49 (7.05–10.23)	0.00	2.07 (1.75–2.44)	0.00	1.94 (1.61–2.33)	0.00	1.88 (1.57–2.26)	0.00
Deaf	12	6.67 (3.43–12.97)	0.00	2.00 (1.11–3.60)	0.09	1.54 (0.78–3.03)_	0.21	1.49 (0.76–2.94)	0.25
Influandpneu death	1,133	Model 1		Model 2		Model 3		Model 4	
Good	673	1 (reference)	0.00	1 (reference)	0.00	1 (reference)	0.00	1 (reference)	0.00
Little trouble	328	3.40 (2.89–4.00)	0.00	1.43 (1.23–1.68)	0.00	1.44 (1.22–1.70)	0.00	1.41 (1.20–1.67)	0.00
A lot of trouble	115	6.84 (5.50–8.51)	0.00	2.17 (1.76–2.67)	0.00	1.79 (1.43–2.25)	0.00	1.70 (1.35–2.14)	0.00
Deaf	17	9.25 (4.63–18.48)	0.00	3.33 (1.86–5.97)	0.00	2.49 (1.25–4.96)	0.01	2.38 (1.20–4.74)	0.01
CLRD death	3,175	Model 1		Model 2		Model 3		Model 4	
Good	1914	1 (reference)	0.00	1 (reference)	0.00	1 (reference)	0.00	1 (reference)	0.00
Little trouble	897	3.30 (3.00–3.62)	0.00	1.44 (1.31–1.58)	0.00	1.29 (1.17–1.42)	0.00	1.26 (1.14–1.39)	0.00
A lot of trouble	331	7.29 (6.34–8.39)	0.00	2.15 (1.88–2.47)	0.00	1.75 (1.52–2.01)	0.00	1.65 (1.44–1.90)	0.00
Deaf	33	7.22 (4.66–11.18)	0.00	2.37 (1.60–3.56)	0.00	1.85 (1.19–2.85)	0.01	1.76 (1.14–2.72)	0.01
Accidents death	2,490	Model 1		Model 2		Model 3		Model 4	
Good	1874	1 (reference)	0.00	1 (reference)	0.00	1 (reference)	0.00	1 (reference)	0.00
Little trouble	462	1.54 (1.36–1.76)	0.00	1.23 (1.09–1.40)	0.03	1.11 (0.94–1.23)	0.11	1.08 (0.94–1.23)	0.27
A lot of trouble	137	2.79 (2.23–3.48)	0.00	1.93 (1.56–2.40)	0.00	1.58 (1.17–1.86)	0.00	1.47 (1.17–1.86)	0.00
Deaf	17	2.82 (1.24–6.44)	0.01	1.97 (0.87–4.34)	0.09	1.69 (0.71–3.64)	0.21	1.61 (0.71–3.64)	0.26

Model 1 unadjusted. Model 2: Adjusted for age. Model 3: Additional adjustment for education, physical activity, smokers, Alcohol intakes, BMI, hypertension, diabetes. Model 4: cancer, stroke, CHD. CVD, cardiovascular disease; CI, confidence interval; HR, hazard ratio; CLRD, chronic lower respiratory disease.

### Subgroup Analyses

In terms of mortality results, a significant interaction was found between all covariates with HL. Compare people with HL diagnosed before the age of 45 vs. after the age of 45, the HL is related to the risk of all-cause mortality to those at the age of >45 years. For all-cause mortality, hearing loss risk at the age of >65 in little trouble, a lot of trouble and deaf were increased by 19% (HR1.19, 95% CI1.16-1.23), 52% (HR1.52, 95% CI 1.46–1.58), and 62% (HR1.62, 95% CI 1.42–1.84), (*p* < 0.001). Adults older than 65 years were associated with the highest all-cause mortality risk in those participants who were deaf. In the subgroup analysis according to these covariates (Table 3), for the same hearing condition, among the older adults (versus young adults), women (versus man), more than high school degreed adults (versus low school degree), <25 kg/m^2^ (versus >25 kg/m^2^), never cigarette (versus former and current smokers), lifetime abstainer (versus former drinker and current drinker) were associated with greatest risk of all-cause mortality.

**TABLE 3:** Subgroup analyses of hearing loss adults for All-Cause Mortality, (United States, 2004–2013).

Subgroups		Good				Little trouble		A lot of trouble		Deaf	
		HR	Lower	Upper	*p*	HR (95%CI)	*p*	HR (95%CI)	*p*	HR (95%CI)	*p*
Age group (years)											
	18–45	1.00				1.02 (0.92–1.13)	0.72	1.13 (0.86–1.50)	0.38	0.73 (0.34–1.58)	0.42
	45–65	1.00				1.06 (1.01–1.11)	0.03	1.17 (1.05–1.30)	0.00	1.36 (1.00–1.86)	0.05
	65-	1.00				1.19 (1.16–1.23)	0.00	1.52 (1.46–1.58)	0.00	1.62 (1.42–1.84)	0.00
Sex											
	Male	1.00				1.13 (1.09–1.16)	0.00	1.36 (1.29–1.43)	0.00	1.54 (1.32–1.80)	0.00
	Female	1.00				1.21 (1.17–1.26)	0.00	1.63 (1.54–1.73)	0.00	1.57 (1.31–1.87)	0.00
Education level											
	Less than high school degree	1.00				1.16 (1.12–1.21)	0.00	1.41 (1.33–1.50)	0.00	1.50 (1.26–1.78)	0.00
	High school degree	1.00				1.16 (1.11–1.21)	0.00	1.46 (1.36–1.57)	0.00	1.50 (1.23–1.84)	0.00
	More than high school degree	1.00				1.17 (1.13–1.22)	0.00	1.49 (1.39–1.60	0.00	1.73 (1.41–2.11)	0.00
BMI (kg/m^2^)											
	<25	1.00				1.21 (1.16–1.25)	0.00	1.50 (1.42–1.59)	0.00	1.57 (1.33–1.86)	0.00
	25–30	1.00				1.16 (1.11–1.21)	0.00	1.44 (1.35–1.53)	0.00	1.58 (1.27–1.97)	0.00
	>30	1.00				1.08 (1.02–1.14)	0.00	1.36 (1.25–1.47)	0.00	1.37 (1.08–1.73)	0.01
Smoking status											
	Never cigarette	1.00				1.23 (1.19–1.28)	0.00	1.64 (1.54–1.75)	0.00	1.53 (1.25–1.88)	0.00
	Former cigarette	1.00				1.13 (1.09–1.17)	0.00	1.39 (1.30–1.47)	0.00	1.67 (1.42–1.96)	0.00
	Current cigarette	1.00				1.09 (1.03–1.15)	0.00	1.21 (1.09–1.34)	0.00	1.15 (0.88–1.51)	0.30
Alcohol intake											
	Lifetime abstainer	1.00				1.21 (1.15–1.27)	0.00	1.56 (1.46–1.66)	0.00	1.51 (1.22–1.86)	0.00
	Former drinker	1.00				1.12 (1.08–1.17)	0.00	1.33 (1.24–1.43)	0.00	1.60 (1.32–1.93)	0.00
	Current drinker	1.00				1.16 (1.12–1.20)	0.00	1.48 (1.39–1.58)	0.00	1.53 (1.24–1.88)	0.00
Physical activity											
	Yes	1.00				1.14 (1.09–1.20	0.00	1.35 (1.23–1.48)	0.00	1.51 (1.18–1.94)	0.00
	No	1.00				1.17 (1.13–1.20)	0.00	1.47 (1.41–1.54)	0.00	1.54 (1.36–1.75)	0.00
Hypertension											
	Yes	1.00				1.15 (1.11–1.19)	0.00	1.41 (1.34–1.48)	0.00	1.49 (1.28–1.72)	0.00
	No	1.00				1.17 (1.12–1.21)	0.00	1.51 (1.42–1.60)	0.00	1.59 (1.35–1.87)	0.00
Diabetes											
	Yes	1.00				1.12 (1.06–1.19)	0.00	1.31 (1.20–1.43)	0.00	1.53 (1.22–1.93)	0.00
	No	1.00				1.17 (1.14–1.20)	0.00	1.49 (1.43–1.56)	0.00	1.55 (1.36–1.76)	0.00
CHD											
	Yes	1.00				1.09 (1.03–1.16)	0.00	1.30 (1.19–1.41)	0.00	1.39 (1.07–1.81)	0.01
	No	1.00				1.17 (1.14–1.21)	0.00	1.50 (1.43–1.57)	0.00	1.59 (1.40–1.80)	0.00
Stroke											
	Yes	1.00				1.09 (1.01–1.16)	0.02	1.24 (1.12–1.37)	0.00	1.35 (1.01–1.83)	0.05
	No	1.00				1.17 (1.14–1.20)	0.00	1.49 (1.43–1.55)	0.00	1.55 (1.37–1.76)	0.00
Cancer											
	Yes	1.00				1.10 (1.05–1.15)	0.00	1.34 (1.24–1.45)	0.00	1.48 (1.16–1.89)	0.00
	No	1.00				1.18 (1.14–1.21)	0.00	1.49 (1.42–1.55)	0.00	1.54 (1.35–1.76)	0.00

Cox proportional hazards regression models were adjusted for sex, age, education, body mass index, smokers, Alcohol intakes, and chronic conditions when appropriate.

### Sensitivity Analyses

In order to confirm the results of the study, we performed two sensitivity analyses. Firstly, participants with chronic diseases had little effect on risk estimates of all-cause mortality were excluded (little trouble: 1.19 (1.16–1.23); a lot of trouble: 1.59 (1.61–1.67); deaf: 1.66 (1.42–1.94). Secondly, all deaths within 2 years of follow-up were excluded [little trouble: 1.17 (1.14–1.20); a lot of trouble: 1.44 (1.38–1.50); deaf: 1.61 (1.43–1.82)] (Supplementary Tables S2A,B).

## Discussion

We used a large number of nationally representative samples of US adults and found that with severity of HL was significantly associated with higher risk of all-cause mortality. Age is an important factor in HL, and the risk of HL increases with age. Participants who reported having little trouble and a lot of trouble in HL had a higher risk for CVD mortality.

Consistent with our studies, most studies have shown that HL in the elderly is correlated with all cause mortality. To our knowledge, these studies as follows: The follow-up of the Norwegian samples rivaled studied association between HL and mortality. A representative Study revealed that HL was associated with a 20% increased mortality risk among 1,958 adults aged 70–79 years [18]. One study showed HL is largely dependent on age and that increased age and extended longevity may increase dramatically in the population that experiences HL [32]. The other study revealed that older adults, aged 80–84 have a higher proportion of HL, and HL largely depends on age [33]. Even after adjusting for other important risk factors, age remains the strongest predictor of HL [34]. In particular, data extracted from the NHANES cohort study showed statistically significant increase for all cause mortality among those a lot of trouble on HL. However, we find this association weakened after adjustment for demographics and chronic disease factors, which was almost the same as that in our study [5]. A previous study also proved that HL is related to mortality, which is consistent with our findings. Appollonio believes that the high mortality risk of HL in the elderly is related to physical health and social function [35]. Because of the serious HL of the elderly, the elderly have a low sense of social participation and social isolation and social loneliness, which accelerate the death risk of the elderly [36]. HL may increase the risk of physical and mental diseases through the physiological system that mediates the response to environmental threats in the elderly and people with severe HL are more likely to develop diseases that may be stress-mediated [37]. Previous studies have shown that hearing aids and cochlear implants are currently effective treatments for the elderly with HL, although patients are often underutilized [38]. However, compared with elderly people who don’t use hearing aids, elderly people who use hearing aids have a much lower chance of depressive symptoms. At the same time, HL usually coexists with tinnitus, so that means that hearing aids amplify external sounds will reduce the perception of tinnitus sounds and related problems [39]. For patients with specific hearing characteristics, hearing aids are beneficial, and the provision of hearing aids will always have the potential to reduce the distress associated with HL [40]. Whether the use of hearing aids and cochlear implants for hearing rehabilitation affects the risk of death in older adults with HL remains uncertain, and further research is needed. This will make “a lot of hearing difficulty” and “profoundly deaf” groups that seem to have about the same mortality rate in many places.

Although the underlying mechanism for the association between HL and mortality is unclear, the following reasons can be considered. The risk factors that increase the risk of death from HL are mainly related to such as smoking, physical activity, occupational noise exposure, and cardiovascular disease and other chronic diseases. Many studies have shown that men have a higher risk of death from HL than women with HL [33, 41]. The main reason is not only that men may be more affected by occupational noise, but also that men have a high smoking rate, which increases the risk of HL [42]. Smoking has always been an important risk factor affecting the health of U.S. adults, and the smoking rate in the United States has also greatly increased [43]. The association between smoking and HL has been confirmed in some other clinical studies [44, 45]. In our study, former smokers increase the risk of mortality with the severity of HL. As lifelong non-smokers sample are 56.5% and the smokers are 43.5% in our study. This sample limits our research on the association between smokers and lifelong non-smoker in HL condition. However, smoking is related to other lifestyles that may adversely affect health. Another study found that participants who do not engage in physical activities have an increased risk of death caused by HL in different hearing levels [46]. As reported previously, severe hearing loss is also associated with slower walking speed [47]. However, as we all know, in the United States and elsewhere, men of all ages have higher incidence and death of CHD than women according to the literature [48, 49]. A previous epidemiological study showed a significant association between elderly patients with HL and CVD mortality [15]. In our study, we can clearly find that without any covariate adjustment, adults with CVD have a higher risk of death than without CVD death. After adjusting the covariates’ results reflect that the HR of deaf patients with CVD risk is reduced. In a few studies, it is demonstrated that HL is related to CHD, and it had a positive association of CHD with the deaf population. Wen Qi Gan et.al. found that exposure to loud occupational noise was significantly associated with the presence of CHD. HL is also a severely indicator for chronic exposure to loud noise [50, 51].

In terms of cause-specific mortality, a similar study from NHIS data found that the association between HL and cause-specific mortality may indirectly increase the risk of mortality due to increased accidental death [52]. However, we did not observe a significant association between HL and accidental death. It was reported that HL is related to cancer events. In addition, another study also analyzed the high mortality of HL patients may also be the existence of chronic diseases [53]. We also adjusted the health status of chronic diseases to modify the effect of HL on mortality in this study. A study on the relationship between increased stroke prevalence and HL in older adults is significant [54]. Some other studies have provided that HL is related to the risk of stroke and stroke-related death. It was observed in one of those studies that adults, age 65 and older, with HL are more likely to suffer from stroke [51, 55]. Our research shows that HL is not only related to all-cause mortality, but also related to each cause-specific mortality. Since HL is a serious threat to the health of people, our research has important public health significance, and more effective measures should be taken to prevent hearing loss.

### Findings of This Study

In our study, in addition to previous studies in which HL was associated with all-cause mortality, we determined whether HL was associated with mortality due to CVD; heart disease; stroke; CLRD; cancer; diabetes; Alzheimer’s disease; influenza and pneumonia; and accidents. Our study confirmed that hearing loss was associated not only with all-cause mortality, but also with specific mortality for each cause. Our subgroup analysis found that for the same hearing condition, in older adults, women, high school education and above, and lifetime alcohol abuse were associated with an increased risk of all-cause mortality, and these important findings could be important for our prevention of HL. Our findings have important public health implications as they suggest that future recommendations could be expanded to preventing or inhibiting the pathogenic factors of hearing loss is important for reducing the risk of death.

Our study has several advantages. First, this study is a prospective cohort study, and the data come from a large sample of representative US adults. NHIS aims to represent the US population and the survey response rate is extremely high (95%–98%). In addition, we constantly adjusted the potential confounding factors in the model and conduct subgroup and sensitivity analysis to ensure the reliability of this study. Some restrictions should be noted. Firstly, the state of HL is obtained self-reported questionnaires of participants, which may cause recall bias. We still cannot completely exclude the remaining confounding factors. 7.5% of the total cohort was excluded (*n* = 37,038/493,036 participants) were excluded owing to missing data on exposures or covariates, as well as hearing loss who are answered by other family members, which might have led to bias if those excluded differed from those not excluded. The results might not be adapted to children. Besides, age available only is a three-level ordinal variable in the US dataset. Secondly, this study did not include non-fatal events, which limited our ability to estimate the risk of incident diseases. Thirdly, I do not have data on the hearing aids and/or cochlear implants available, poor communication between doctors and hearing loss patients results in a variety of adverse outcomes.

### Conclusion

In the present study, elderly participants with extreme HL (deaf patients) are associated with increased risk of all-cause and cause-specific mortality. The findings of our research and the growing literature have also revealed the potential burden of HL and consequences, and it is necessary to improve the screening and management of HL among U.S. adults, especially among the elderly.

## Data Availability

The NHIS data are available from www.cdc.gov/nchs/nhis/index.htm.
