# Supplementary material for: Association of all Cause and Cause-Specific Mortality With Hearing Loss Among US Adults: A Secondary Analysis Study
Source: Int J Public Health. 2022 May 17;67:1604785. doi: 10.3389/ijph.2022.1604785 (PMC9151924; doi:10.3389/ijph.2022.1604785)
Supplement: Supplementary file 1 [file DataSheet1.docx]

| **Supplementary Table A** International classification of diseases, 10th revision was used in study, the United States, 2004-2013. | | | |
| --- | --- | --- | --- |
| Cardiovascular disease—I00-I09, I11, I13, I20-I51, I60-I69 | | | |
| Cancer—C00-C97 |  |  |  |
| Diabetes—E10-E14 |  |  |  |
| Chronic lower respiratory diseases—J40-J47 | | |  |
| Influenza and pneumonia—J09-18 | |  |  |
| Alzheimer’s disease—G30 |  |  |  |
| Accidents —V01-X59 |  |  |  |
| ICD-10=international classification of diseases, 10th revision. | | | |

**Supplementary Table 2A** Participants with chronic diseases, the United States, 2004-2013.

| **All cause** | HR | Lower | Upper | P |
| --- | --- | --- | --- | --- |
|  |  |  |  |  |
| Good | 1.00 |  |  | 0.00 |
| Little trouble | 1.19 | 1.16 | 1.23 | 0.00 |
| A lot of trouble | 1.59 | 1.51 | 1.67 | 0.00 |
| Deaf | 1.66 | 1.42 | 1.94 | 0.00 |

*participates with chronic diseases had little effect on risk estimates of all-cause mortality were excluded

**Supplementary Table 2B** All deaths within two years of follow-up, the United States, 2004-2013.

| **All cause** | HR | Lower | Upper | P |
| --- | --- | --- | --- | --- |
|  |  |  |  |  |
| Good | 1.00 |  |  | 0.00 |
| Little trouble | 1.17 | 1.14 | 1.20 | 0.00 |
| A lot of trouble | 1.44 | 1.38 | 1.50 | 0.00 |
| Deaf | 1.61 | 1.43 | 1.82 | 0.00 |

*All deaths within two years of follow-up were excluded
